# Supplementary material for: Genome-wide meta-analysis associates HLA-DQA1/DRB1 and LPA and lifestyle factors with human longevity
Source: Nat Commun. 2017 Oct 13;8:910. doi: 10.1038/s41467-017-00934-5 (PMC5715013; doi:10.1038/s41467-017-00934-5)
Supplement: Supplementary file 1 — Description of additional supplementary files [file 41467_2017_934_MOESM1_ESM.pdf]

Description of Additional Supplementary Files

File Name: Supplementary Data 1
Description: Cohort characteristics.

File Name: Supplementary Data 2
Description: Summary Statistics for each cohort.

File Name: Supplementary Data 3
Description: Lookup of previously reported longevity associations in present lifespan study. Chr – Chromosome, Position - position on build 37.3, OR - Odds ratio of allele in long-lived cases vs controls, P1 - P-value reported by cited source, N - the total number of parent lifespans analysed, HR a1 - the Cox hazard ratio on self for 1 copy of a1, Years - the mean effect on lifespan for 1 copy of a1, direction of the effect is denoted by the sign, P2 - the p-value in present study for the hypothesis that there is no association between the SNP and mortality, two sided t test, Extrapolated OR– the estimated OR derived from the LifeGen hazard ratio and the relationship between OR and HR at APOE, * - the highlighted studies followed a candidate gene approach and therefore their p-value threshold is >>5E-08

File Name: Supplementary Data 4
Description: Previous association reports for the four genome-wide significant loci, acquired through PhenoScanner.

File Name: Supplementary Data 5
Description: Genetic Correlations with Mortality and 113 traits, measured using LDHub and LDScore regression. Results from the genetic correlation analysis conducted using the LDhub web server (http://ldsc.broadinstitute.org/). rg represents the genetic correlation value, se the standard error and p the p value. Finally, PMID refers to the Pubmed ID of the paper reporting the results of the GWAS used for the analysis. Results for Breast Cancer, CRP, Systolic Blood Pressure (SBP), Diastolic Blood Pressure (DBP) were estimated locally using the ldsc software as they are not available on the ldsc web server.

File Name: Supplementary Data 6
Description: Studies used for MR Study.
