# Supplementary material for: Genome-wide meta-analysis associates HLA-DQA1/DRB1 and LPA and lifestyle factors with human longevity
Source: Nat Commun. 2017 Oct 13;8:910. doi: 10.1038/s41467-017-00934-5 (PMC5715013; doi:10.1038/s41467-017-00934-5)
Supplement: Supplementary file 2 — Supplementary Information [file 41467_2017_934_MOESM2_ESM.pdf]

Supplementary Figure 1 | Locus zoom plot of the p-values of association for 1 further genome-wide significant association when meta-analysing CHAREG EU's longevity GWAMA and LifeGen across both parents in cohorts of European Ancestry. By combining our discovery association analysis with a long-livedness GWAS, we found an association with longevity for rs1919453, between genes AKAP7 and EBP41L2, but had no further data with which to seek replication. In the absence of replication, it is unclear if this SNP is reliably associated with lifespan.

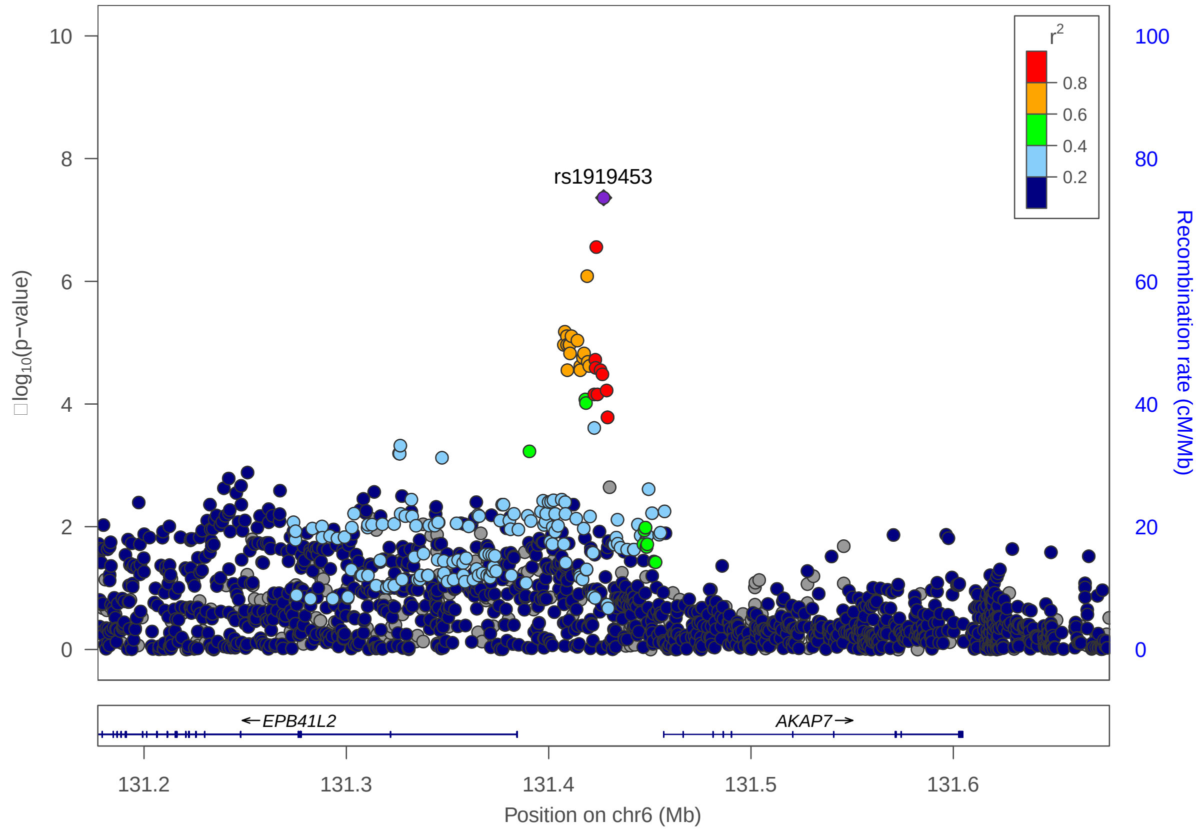

<u>Supplementary Figure 2 | Locus zoom plots for chromosome 2 region.</u> African mothers (**A**), African fathers (**B**), and Europeans with both parents combined (**C**). rs10198124 shows consistent association with lifespan in African fathers across cohorts, but no evidence in African mothers or Europeans of both ancestries. (**D**) The forest plot shows estimates of the hazard ratio for carrying 1 copy of the risk allele +/- 2 SE, for each cohort, i.e. observed father-child effect sizes were doubled. The apparent association with lifespan, in African fathers may be due to chance, as there are not even suggestive associations in African mothers or either European parent.

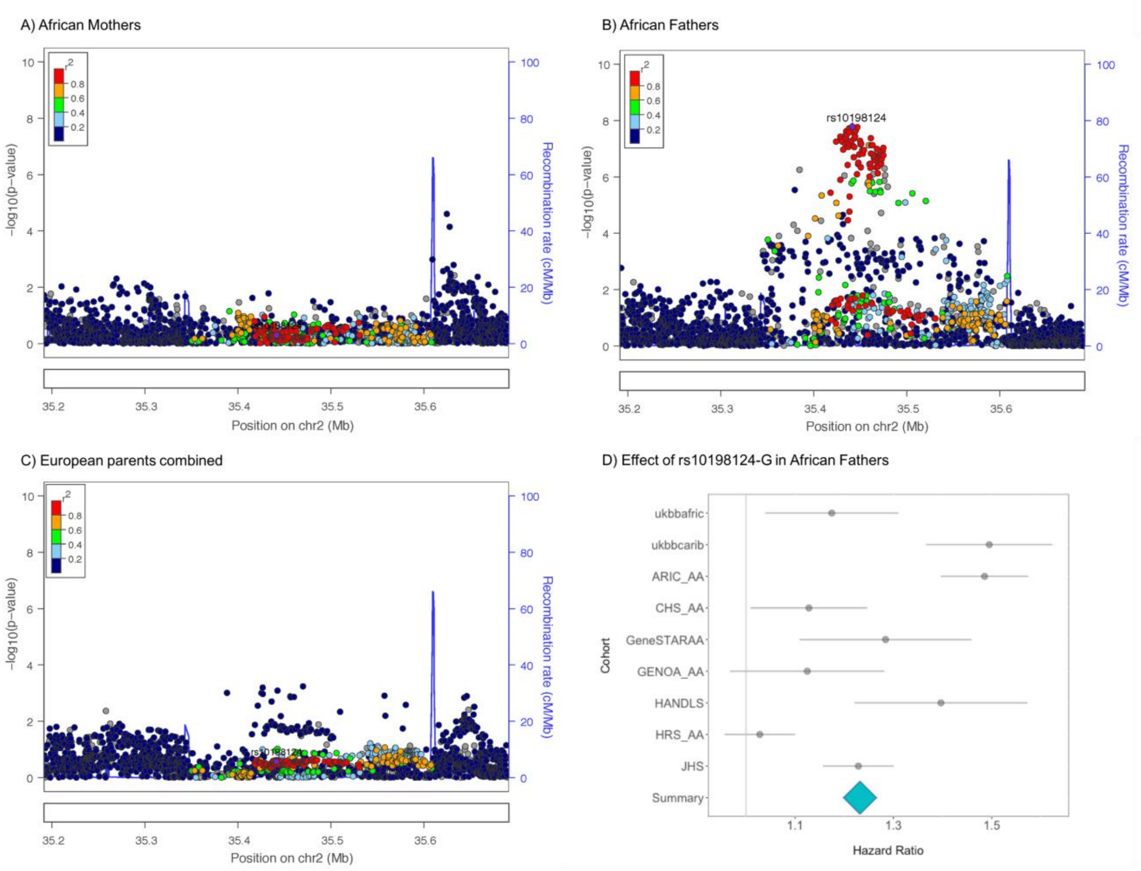

Supplementary Figure 3 | Locus Zoom plots of the Validated Candidate Loci.

GWAMA and LifeGen across both parents in cohorts of European Ancestry.

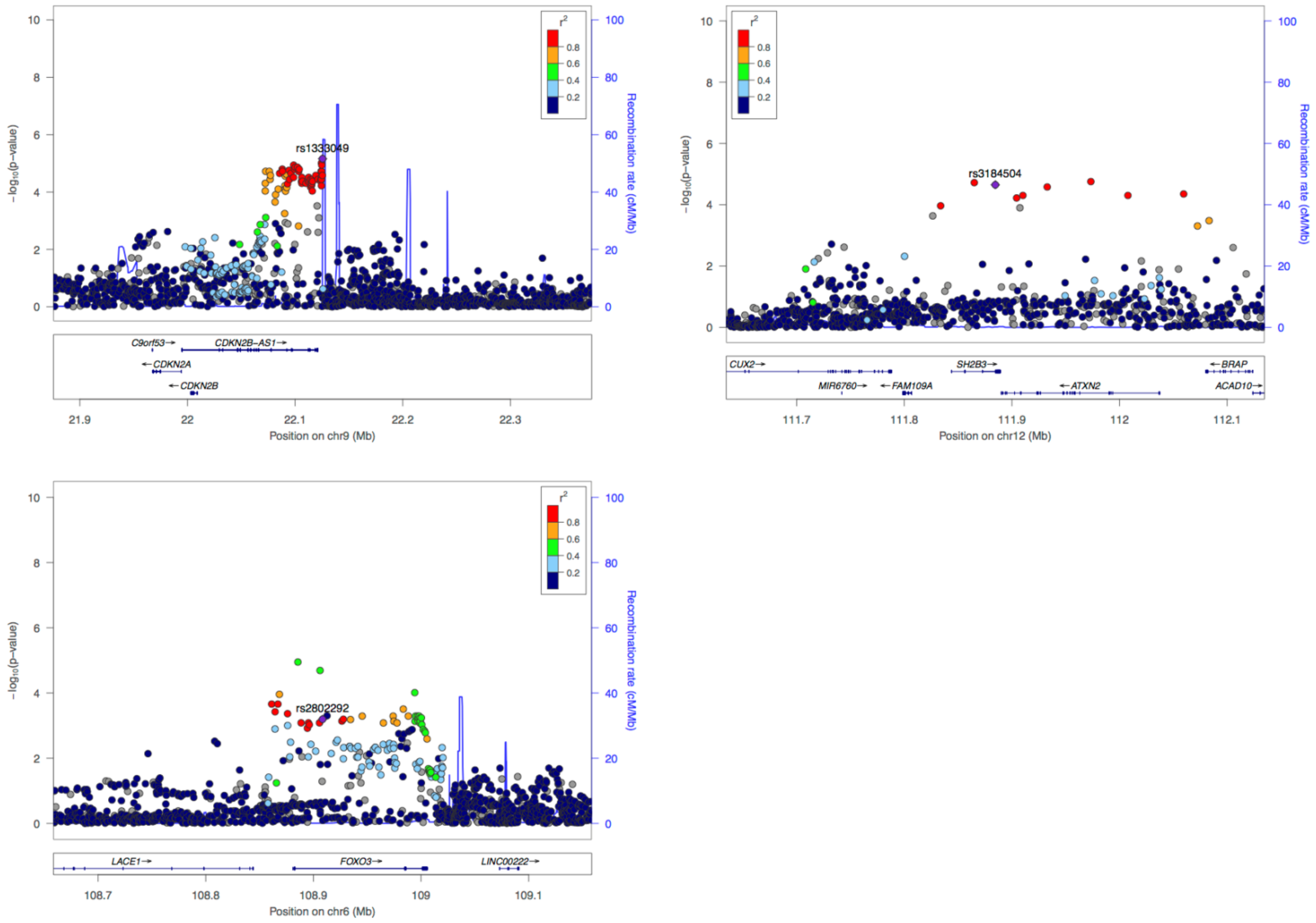

Supplementary Figure 4 | Meaningful genetic correlations (r_g) between and mortality and trait. Traits which genetically correlate with increased mortality are shown in red and those correlating with increased longevity are in green. 113 traits were tested but only statistically significant correlations (FDR < 5%) with $|r_g| > 0.15$ are shown. Traits that are similar are all shown, for completeness and to avoid selection bias. The remaining traits and the studies from which the displayed genetic correlations were sourced can be found in Supplementary Table 3. HOMA-IR; homeostatic model assessment of insulin resistance, SBP; systolic blood pressure, CRP; C-reactive protein, DBP; diastolic blood pressure, HbA1c; glycated haemoglobin A1, HDL; heavy density lipoprotein.

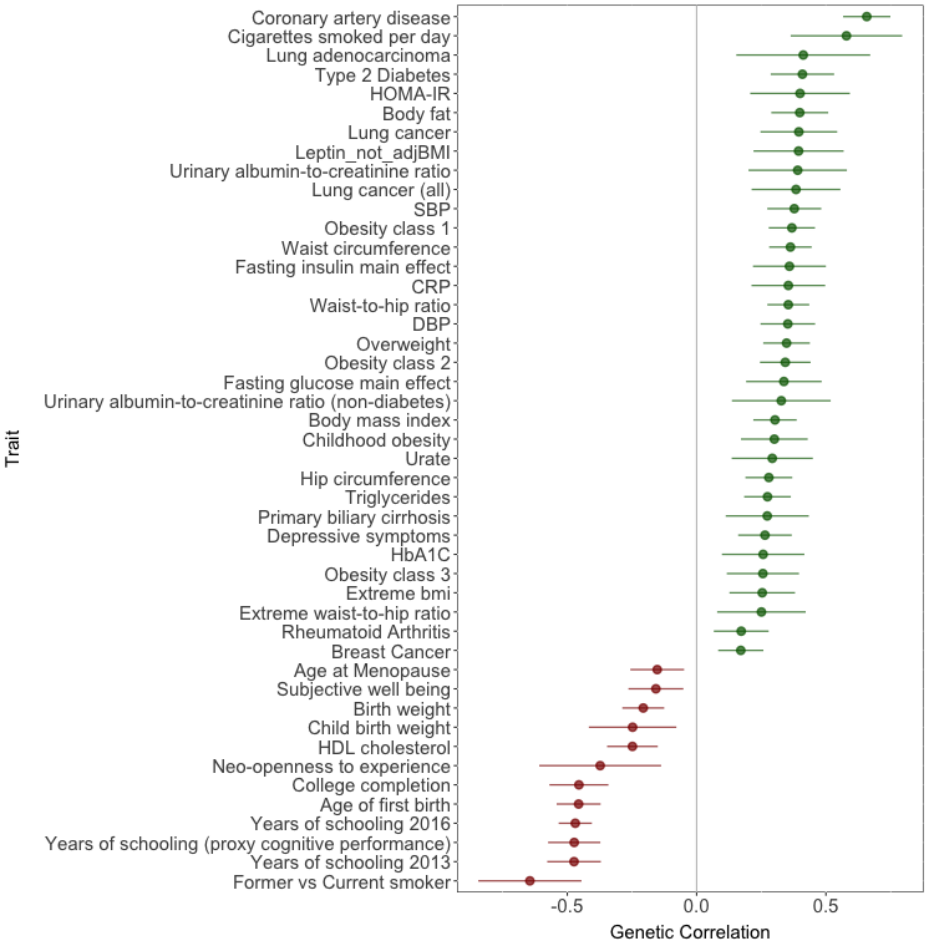

Supplementary Table 1 | Genetic correlations and partial correlations of twelve trait clusters with mortality. The genetic correlation (rg) between the trait and lifespan was calculated from summary statistics using LD Score regression. The partial correlations (Partial rg), were derived from the genetic correlations using the matrix-inversion method. The lifespan trait is the LifeGen GWAMA for Europeans, both parents combined. CAD; coronary artery disease, T2D; type 2 diabetes, DL/WHR; dyslipidaemia/waist-hip-ratio, BP; blood pressure, Kidney; kidney function, BC; breast cancer, RA; rheumatoid arthritis, AM; age of menarche.

	rg	Partial rg
Smoking	0.68	0.61
CAD	0.66	0.62
T2D	0.48	0.33
DL/WHR	0.41	-0.19
BP	0.39	0.16
Obesity	0.37	0.24
Kidney	0.33	0.34
BC	0.17	0.23
RA	0.17	0.09
AM	-0.15	-0.01
Happiness	-0.24	-0.19
Edu	-0.5	-0.18

Supplementary Table 2 | Results from the discovery step MR for the 28 traits that were significant, prior to removal of trait overlap.

Exposure	# snps	b	se	pval	pleio_p	qvalue	het_p
Omega-6 fatty acids	13	0.0334	0.0135	1.32E-02	0.0203	3.80E-02	5.49E-03
LDL cholesterol	78	0.0543	0.0091	2.80E-09	0.8981	3.73E-08	1.13E-08
Obesity class 1	18	0.0435	0.0066	4.76E-11	0.2723	9.52E-10	4.95E-01
Alzheimer's disease	20	0.0181	0.0064	4.59E-03	0.725	1.53E-02	3.33E-01
HDL cholesterol	85	-0.0448	0.0127	4.07E-04	0.1318	1.55E-03	3.56E-14
Total cholesterol	85	0.0554	0.0096	9.41E-09	0.386	9.06E-08	2.75E-09
Coronary heart disease	39	0.0806	0.0109	1.67E-13	0.926	1.34E-11	6.10E-12
Extreme body mass index	7	0.0257	0.0063	4.08E-05	0.882	1.92E-04	2.92E-01
Myocardial infarction	25	0.0643	0.011	5.80E-09	0.1206	6.63E-08	1.16E-05
Omega-3 fatty acids	6	0.0302	0.0122	1.33E-02	0.5887	3.80E-02	5.28E-01
Years of schooling	69	-0.1774	0.0266	2.69E-11	0.0139	7.18E-10	1.37E-03
DBP	9	0.0144	0.0028	3.94E-07	0.1805	2.87E-06	1.99E-01
Hip circumference	52	0.0976	0.0218	7.86E-06	0.7006	4.49E-05	3.41E-07
Type 2 diabetes	25	0.0175	0.0069	1.15E-02	0.178	3.64E-02	2.20E-01
CRP	48	-0.041	0.0109	1.67E-04	0.9083	7.03E-04	3.31E-15
Obesity class 3	2	0.0237	0.0065	2.58E-04	NA	1.03E-03	1.00E+00
Apolipoprotein B	19	0.0535	0.0093	1.02E-08	0.5418	9.06E-08	3.57E-02
Body mass index	78	0.1226	0.0173	1.26E-12	0.3865	5.03E-11	1.01E-05
Triglycerides	54	0.0326	0.0129	1.18E-02	0.2659	3.64E-02	3.66E-06
Fasting insulin	14	0.1619	0.0501	1.23E-03	0.6666	4.29E-03	8.65E-01
SBP	8	0.0088	0.002	1.12E-05	0.0341	5.98E-05	2.16E-01
Obesity class 2	11	0.0335	0.0059	1.68E-08	0.9266	1.35E-07	3.67E-01
Childhood obesity	10	0.036	0.0109	1.00E-03	0.0475	3.64E-03	2.93E-05
Squamous cell lung cancer	3	0.0494	0.0098	4.86E-07	0.3085	3.24E-06	4.06E-01
Breast Cancer	111	0.0155	0.0039	6.65E-05	0.5674	2.95E-04	3.45E-02
Overweight	14	0.0592	0.0125	2.25E-06	0.3002	1.38E-05	1.45E-01
Cigarettes smoked per day	rs12914385	0.0169	0.0027	6.47E-10	NA	1.03E-08	NA
Ischaemic stroke	rs4984814	0.0058	0.0013	1.39E-05	NA	6.97E-05	NA

Supplementary Table 3 | Number of SNPs which composed the Instrumental Variables before and after pruning

Trait	Initial N SNPs	LD pruned SNPs
Alzheimer's disease	20	18
Apolipoprotein B	21	3
Breast Cancer	134	131
Body mass index SD (kg/m^2)	79	66
Cigarettes smoked per day	1	1
Coronary heart disease	41	28
CRP	53	44
DBP	9	3
Fasting insulin log pmol/L	14	6
HDL cholesterol SD (mg/dL)	89	40
Ischaemic stroke	1	1
LDL cholesterol SD (mg/dL)	80	17
Omega-3 fatty acids SD	6	1
SBP	11	4
Squamous cell lung cancer	4	3
Total cholesterol SD (mg/dL)	88	12
Triglycerides SD (mg/dL)	54	18
Type 2 diabetes	34	29
Years of schooling SD (years)	73	67

Supplementary Note 1 | Cohort and Consortium Acknowledgements

ACPRC was supported by Biotechnology and Biological Sciences Research Council grant BBFO22441/1.
The ARIC study is carried out as a collaborative study supported by National Heart, Lung, and Blood Institute contracts (HHSN268201100005C, HHSN268201100006C, HHSN268201100007C, HHSN268201100008C, HHSN268201100009C, HHSN268201100010C, HHSN268201100011C, and HHSN268201100012C), R01HL087641, R01HL59367 and R01HL086694; National Human Genome Research Institute contract U01HG004402; and National Institutes of Health contract HHSN268200625226C. The authors thank the staff and participants of the ARIC study for their important contributions. Infrastructure was partly supported by Grant Number UL1RR025005, a component of the National Institutes of Health and NIH Roadmap for Medical Research. The study was funded in part by R56 DK104806-01A1 and R21HL123677-01 to NF R01-DK089256 (North), N01-HC-55015, N01-HC-55016, N01-C-55018, N01-HC-55019, N01-HC-55020, N01-HC-55021, N01-HC-55022, R01HL087641, R01HL59367, R01HL086694, U01HG004402, HHSN268200625226C, UL1RR025005, UL1 RR 025005. Contents are solely the responsibility of the authors and do not necessarily represent the official view of NCRR or NIH.
The Austrian Stroke Prevention Family Study: The research reported in this article was funded by the Austrian Science Fund (FWF) grants number PI904, P20545-P05 and P13180. The Medical University of Graz and the Steiermärkische Krankenanstalten Gesellschaft support the databank of the ASPS-Fam. The authors thank the staff and the participants of the ASPS-Fam for their valuable contributions. The authors thank Birgit Reinhart for her long-term administrative commitment and Ing Johann Semmler for the technical assistance at creating the DNA bank.
Berlin Aging Study II (BASE-II) was supported by the German Federal Ministry of Education and Research (BMBF [grants #16SV5536K, #16SV5537, #16SV5538, and #16SV5837; previously #01UW0808]). Additional contributions (e.g. financial, equipment, logistics, personnel) are made from each of the other participating sites, i.e. the Max Planck Institute for Human Development (MPIB), Max Planck Institute for Molecular Genetics (MPIMG), Charite University Medicine, German Institute for Economic Research (DIW), all located in Berlin, Germany, and University of Lübeck in Lübeck, Germany. Financial support (all to C. Lill) by grants from the Renate Maaß foundation, the German Research Foundation (FOR2488/1, GZ LI 2654/2-1) and the University of Lübeck (medical section, grant number: J21-2016).
Cardiovascular Health Study: This CHS research was supported by NHLBI contracts HHSN268201200036C, HHSN268200800007C, HHSN268200960009C, N01HC55222, N01HC85079, N01HC85080, N01HC85081, N01HC85082, N01HC85083, N01HC85086; and NHLBI grants U01HL080295, R01HL085251, R01HL087652, R01HL105756, R01HL103612, R01HL120393, and R01HL130114 with additional contribution from the National Institute of Neurological Disorders and Stroke (NINDS). Additional support was provided through R01AG023629 from the National Institute on Aging (NIA). A full list of principal CHS investigators and institutions can be found at CHS-NHLBI.org. The provision of genotyping data was supported in part by the National Center for Advancing Translational Sciences, CTSI grant UL1TR000124, and the National Institute of Diabetes and Digestive and Kidney Disease Diabetes Research Center (DRC) grant DK063491 to the Southern California Diabetes Endocrinology Research Center. The content is solely the responsibility of the authors and does not necessarily represent the official views of the National Institutes of Health. B Psaty serves on the DSMB of a clinical trial funded by the manufacturer (Zoll LifeCor) and on the Steering Committee of the Yale Open Data Access Project funded by Johnson & Johnson.
CILENTO was supported by PON03PE_00060_7 grant from the Italian Ministry of Universities and Research, Interomics Flagship grant from CNR, Cilento study grant from Assessorato Ricerca Regione Campania, 2011-PDR-13 grant from the Fondazione con il SUD.
The CoLaus study was and is supported by research grants from GlaxoSmithKline, the Faculty of Biology and Medicine of Lausanne, and the Swiss National Science Foundation (grants 33CSCO-122661, 33CS30-139468, and 33CS30-148401).
The ECGUT study was funded by Estonian Research Council Grant IUT20-60 and PUT1660, EU H2020 grant 692145, and European Union through the European Regional Development Fund (Project No. 2014-2020.4.01.15-0012) GENTRANSMED.

The ERF study as a part of EUROSPAN (European Special Populations Research Network) was supported by European Commission FP6 STRP grant number 018947 (LSHG-CT-2006-01947) and also received funding from the European Community's Seventh Framework Programme (FP7/2007-2013)/grant agreement HEALTH-F4-2007-201413 by the European Commission under the programme "Quality of Life and Management of the Living Resources" of 5th Framework Programme (no. QLG2-CT-2002-01254). The ERF study was further supported by ENGAGE consortium and CMSB. High-throughput analysis of the ERF data was supported by a joint grant from Netherlands Organisation for Scientific Research and the Russian Foundation for Basic Research (NWO-RFBR 047.017.043). Exome sequencing in ERF was supported by the ZonMw grant (project 91111025). We are grateful to all study participants and their relatives, general practitioners and neurologists for their contributions and to P. Veraart for her help in genealogy, J. Vergeer for the supervision of the laboratory work and P. Snijders for his help in data collection.
GeneSTAR was supported by grants from the National Institutes of Health/National Heart, Lung, and Blood Institute (U01 HL72518, HL087698, HL49762, HL59684, HL58625, HL071025, HL092165, HL099747), National Institutes of Health/National Institute of Nursing Research (NR0224103, NR008153), National Institutes of Health/National Institute of Neurological Disorders and Stroke (NS062059) and by a grant from the National Institutes of Health/National Center for Research Resources (M01-RR000052) to the Johns Hopkins General Clinical Research Center.
GENOA (Genetic Epidemiology Network of Arteriopathy): Support for GENOA was provided by the National Heart, Lung and Blood Institute (HL054464, HL054457, HL054481, HL071917, HL87660, HL119443) of the National Institutes of Health. Genotyping was performed at the Mayo Clinic. We thank Eric Boerwinkle, PhD and Megan L. Grove from the Human Genetics Center and Institute of Molecular Medicine and Division of Epidemiology, University of Texas Health Science Center - Houston, for their help with genotyping. We would also like to thank the families that participated in the GENOA study.
Genotyping of the GS:SFHS samples was carried out by the Edinburgh Clinical Research Facility, University of Edinburgh and was funded by the Medical Research Council UK and the Wellcome Trust (Wellcome Trust Strategic Award “STratifying Resilience and Depression Longitudinally” (STRADL) (Reference 104036/Z/14/Z). GS20K received core support from the Chief Scientist Office of the Scottish Government Health Directorates [CZD/16/6] and the Scottish Funding Council [HR03006]. The MRC provides core funding to the QTL in Health and Disease research program at the MRC HGU, IGMM, University of Edinburgh.
The Healthy Aging in Neighborhoods of Diversity across the Life Span (HANDLS) study was supported by the Intramural Research Program of the National Institute on Aging, National Institutes of Health (project # Z01-AG000513 and human subjects protocol number 09-AG-N248). The authors would like to thank study participants and the HANDLS medical staff. The authors also would like to thank Dr. Michael A. Nalls for their help in variant calling and genotype quality control. Data analyses for the HANDLS study utilized the high-performance computational resources of the Biowulf Linux cluster at the National Institutes of Health, Bethesda, Maryland, USA (http://hpc.nih.gov).
HRS is supported by the National Institute on Aging (NIA U01AG009740). Genotyping was funded separately by NIA (RC2 AG036495, RC4 AG039029). Our genotyping was conducted by the NIH Center for Inherited Disease Research (CIDR) at Johns Hopkins University. Genotyping quality control and final preparation of the data were performed by the Genetics Coordinating Center at the University of Washington.
The iPSYCH project is funded by the Lundbeck Foundation, the universities and university hospitals of Aarhus and Copenhagen and Statens Staten Serums Institute (R155-2014-1724 & R102-A9118). Y WANG: YW is funded through the FRIPRO Mobility Grant, contract no 251134 as part of EU7 Marie Curie grant no 608695.
We thank the Jackson Heart Study (JHS) participants and staff for their contributions to this work. The JHS is supported by contracts HHSN268201300046C, HHSN268201300047C, HHSN268201300048C, HHSN268201300049C, HHSN268201300050C from the National Heart, Lung, and Blood Institute and the National Institute on Minority Health and Health Disparities. Dr. Wilson is supported by U54GM115428 from the National Institute of General Medical Sciences.

For the MICROS study, we thank the primary care practitioners and the personnel of the Hospital of Silandro (Department of Laboratory Medicine) for their participation and collaboration in the research project. In South Tyrol, the study was supported by the Ministry of Health and Department for Innovation, Universities and Research of the Autonomous Province of Bolzano, South Tyrol, the South Tyrolean Sparkasse Foundation, and the European Union framework program 6 EUROSPAN project (contract no. LSHG-CT-2006-018947).
The Nijmegen Biomedical Study is a population-based survey conducted at the Department for Health Evidence and the Department of Laboratory Medicine of the Radboud university medical center. The set-up of NBS was financed by the Radboudumc, the municipality Nijmegen and the community health service Nijmegen. Principal investigators of the Nijmegen Biomedical Study are L.A.L.M. Kiemeney, A.L.M. Verbeek, D.W. Swinkels and B. Franke.
NSHD are very grateful to the members of this birth cohort for their continuing interest and participation in the study. This work was funded by the Medical Research Council [MC_UU_12019/1]. Data used in this publication are available to bona fide researchers upon request to the NSHD Data Sharing Committee via a standard application procedure. Further details can be found at http://www.nshd.mrc.ac.uk/data. doi: 10.5522/NSHD/Q101.
The NTR study was supported by multiple grants from the Netherlands Organization for Scientific Research (NWO: 016-115-035, 463-06-001, 451- 04-034); ZonMW (31160008, 911-09-032); and NWO 480-15-001/674: Netherlands Twin Registry Repository: researching the interplay between genome and environment; The Amsterdam Public Health Institute (APH) and Neuroscience Campus Amsterdam (NCA); Biomolecular Resources Research Infrastructure (BBMRI–NL, 184.021.007), European Research Council (ERC-230374); Genotyping was made possible by grants from NWO/SPI 56-464-14192, Genetic Association Information Network (GAIN) of the Foundation for the National Institutes of Health, Rutgers University Cell and DNA Repository (NIMH U24 MH068457-06), the Avera Institute, Sioux Falls (USA) and the National Institutes of Health (NIH R01 HD042157-01A1, MH081802, Grand Opportunity grants 1RC2 MH089951 and 1RC2 MH089995).
OGP was suppoted by a a grant from the Italian Ministry of Education, University and Research (MIUR) n°: 5571/DSPAR/2002.
ORCADES was supported by the Chief Scientist Office of the Scottish Government (CZB/4/276, CZB/4/710), the Royal Society, the MRC Human Genetics Unit, Arthritis Research UK and the European Union framework program 6 EUROSPAN project (contract no. LSHG-CT-2006-018947). DNA extractions were performed at the Wellcome Trust Clinical Research Facility in Edinburgh. We would like to acknowledge the invaluable contributions of the research nurses in Orkney, the administrative team in Edinburgh and the people of Orkney.
The PROSPER study was supported by an investigator initiated grant obtained from Bristol-Myers Squibb. Prof. Dr. J. W. Jukema is an Established Clinical Investigator of the Netherlands Heart Foundation (grant 2001 D 032). Support for genotyping was provided by the seventh framework program of the European commission (grant 223004) and by the Netherlands Genomics Initiative (Netherlands Consortium for Healthy Aging grant 050-060-810).
The Australian Research Council (FT110100548) and Australian National Health and Medical Research Council (APP1103623). NHMRC and NIH funding for phenotype collection and genotyping.
The generation and management of GWAS genotype data for the Rotterdam Study (RS I, RS II) was executed by the Human Genotyping Facility of the Genetic Laboratory of the Department of Internal Medicine, Erasmus MC, Rotterdam, The Netherlands. The GWAS datasets are supported by the Netherlands Organisation of Scientific Research NWO Investments (nr. 175.010.2005.011, 911-03-012), the Genetic Laboratory of the Department of Internal Medicine, Erasmus MC, the Research Institute for Diseases in the Elderly (014-93-015; RIDE2), the Netherlands Genomics Initiative (NGI)/Netherlands Organisation for Scientific Research (NWO) Netherlands Consortium for Healthy Aging (NCHA), project nr. 050-060-810. We thank Pascal Arp, Mila Jhamai, Marijn Verkerk, Lizbeth Herrera and Marjolein Peters, PhD, and Carolina Medina-Gomez, MSc, for their help in creating the GWAS database, and Karol Estrada, PhD, Yurii Aulchenko, PhD, and Carolina Medina-Gomez, PhD, for the creation and analysis of imputed data. The Rotterdam Study is funded by Erasmus Medical Center and Erasmus University, Rotterdam,

Netherlands Organization for the Health Research and Development (ZonMw), the Research Institute for Diseases in the Elderly (RIDE), the Ministry of Education, Culture and Science, the Ministry for Health, Welfare and Sports, the European Commission (DG XII), and the Municipality of Rotterdam. The authors are grateful to the study participants, the staff from the Rotterdam Study and the participating general practitioners and pharmacists.
The Viking Health Study – Shetland (VIKING) was supported by the MRC Human Genetics Unit quinquennial programme grant “QTL in Health and Disease”. DNA extractions and genotyping were performed at the Edinburgh Clinical Research Facility, University of Edinburgh. We would like to acknowledge the invaluable contributions of the research nurses in Shetland, the administrative team in Edinburgh and the people of Shetland.
The Vitality 90+ study has been financially supported by the Academy of Finland (grants 286284 (T.L), 132704 (M.H.), and 250602 (Coctel to M.J.); the Finnish Foundation of Cardiovascular Research (T.L.); the Tampere Tuberculosis Foundation (T.L., M.H.); the Emil Aaltonen Foundation (T.L); the Finnish Cultural Foundation, Pirkanmaa Regional fund (N.M); and the Yrjö Jahnsson Foundation (T.L., M.H.) This work was also supported by grants from the Competitive Research Fund of Pirkanmaa Hospital District (9M017, 9N013 to M.H, 9P002 to M.J. and X51001 for T.L.).
The WGHS is supported by the National Heart, Lung, and Blood Institute (HL043851 and HL080467) and the National Cancer Institute (CA047988 and UM1CA182913) with funding for genotyping provided by Amgen.
BCAC is funded by Cancer Research UK [C1287/A16563, C1287/A10118], the European Union's Horizon 2020 Research and Innovation Programme (grant numbers 634935 and 633784 for BRIDGES and B-CAST respectively), and by the European Community´s Seventh Framework Programme under grant agreement number 223175 (grant number HEALTH-F2-2009-223175) (COGS). Funding for the iCOGS infrastructure came from: the European Community's Seventh Framework Programme under grant agreement n° 223175 (HEALTH-F2-2009-223175) (COGS), Cancer Research UK (C1287/A10118, C1287/A 10710, C12292/A11174, C1281/A12014, C5047/A8384, C5047/A15007, C5047/A10692, C8197/A16565), the National Institutes of Health (CA128978) and Post-Cancer GWAS initiative (1U19 CA148537, 1U19 CA148065 and 1U19 CA148112 - the GAME-ON initiative), the Department of Defence (W81XWH-10-1-0341), the Canadian Institutes of Health Research (CIHR) for the CIHR Team in Familial Risks of Breast Cancer, Komen Foundation for the Cure, the Breast Cancer Research Foundation, and the Ovarian Cancer Research Fund.
